# Multiple ecosystem services in a working landscape

**DOI:** 10.1371/journal.pone.0166595

**Published:** 2017-03-16

**Authors:** Danny J. Eastburn, Anthony T. O’Geen, Kenneth W. Tate, Leslie M. Roche

**Affiliations:** 1 Department of Plant Sciences, University of California Davis, Davis, California, United States of America; 2 Department of Land, Air, and Water Resources, University of California Davis, Davis, California, United States of America; Tennessee State University, UNITED STATES

## Abstract

Policy makers and practitioners are in need of useful tools and models for assessing ecosystem service outcomes and the potential risks and opportunities of ecosystem management options. We utilize a state-and-transition model framework integrating dynamic soil and vegetation properties to examine multiple ecosystem services—specifically agricultural production, biodiversity and habitat, and soil health—across human created vegetation states in a managed oak woodland landscape in a Mediterranean climate. We found clear tradeoffs and synergies in management outcomes. Grassland states maximized agricultural productivity at a loss of soil health, biodiversity, and other ecosystem services. Synergies existed among multiple ecosystem services in savanna and woodland states with significantly larger nutrient pools, more diversity and native plant richness, and less invasive species. This integrative approach can be adapted to a diversity of working landscapes to provide useful information for science-based ecosystem service valuations, conservation decision making, and management effectiveness assessments.

## Introduction

For over a decade, science and policy efforts have increasingly focused on valuing and preserving ecosystem services—the collective benefits humanity obtains from the environment [1]. Globally, ecosystem service payments and investment programs are seeing tremendous growth [2–4], and receiving increasing support from a diversity of scientists, environmental groups, land managers, government agencies, and non-governmental organizations [2, 5–8]. Of course, considerable challenges—including climate change, increasing resource needs driven by growing human population, changing fire regimes and land use conversion—remain for delivering ecosystem services [8–10]. For working landscapes such as grazed rangelands and managed forest lands, there are two high priority areas: 1) linking management decisions with on-the-ground measures of ecosystem service outcomes; and 2) quantifying tradeoffs and synergies among multiple ecosystem services under different land management options [2, 9, 11–13].

Policy makers and practitioners need useful insight and practical advice generated from tools and models that assess the potential risks and benefits of ecosystem management options. Over the past thirty years, state and transition models (STM) have received increasing attention as potentially viable frameworks for applied ecological science and management [14, 15]. STMs are one way of describing the multiple states and associated ecosystem services that a particular site can achieve; thresholds and ecological resilience of individual states to stressors; and the underlying roles of management practices and natural processes in pushing a site between states [16]. STMs have tremendous potential in adaptive management approaches for complex systems and are positioned to become one of the most extensive and utilized land management frameworks in the world [17, 18].

**Fig 1:**
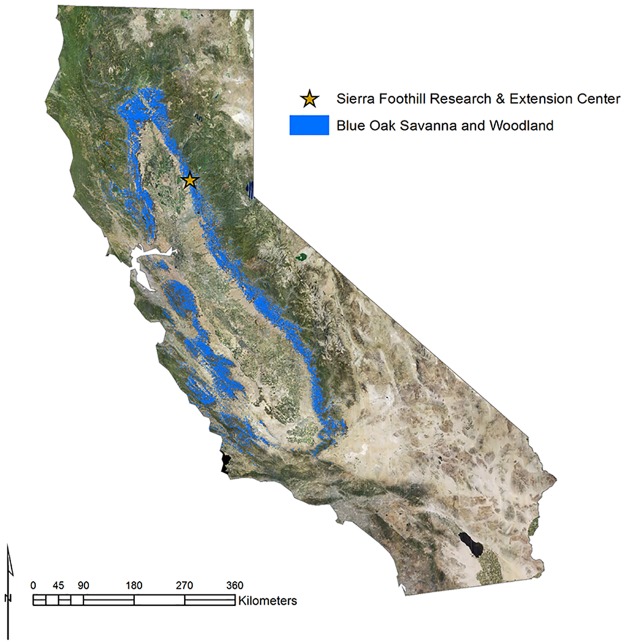
California’s blue oak woodland range. California’s endemic blue oak (*Quercus douglasii*) woodlands and savannas span approximately 4 million hectares. Our analysis focused on a managed oak woodland landscape at the University of California Sierra Foothill Research and Extension Center (SFREC) in Yuba County, California, US. Map was created using data from California Department of Forestry and Fire Protection FRAP 2006. The digital orthoimagery was acquired from United States Department of Agriculture National Geospatial Data Asset NAIP Imagery.

**Fig 2:**
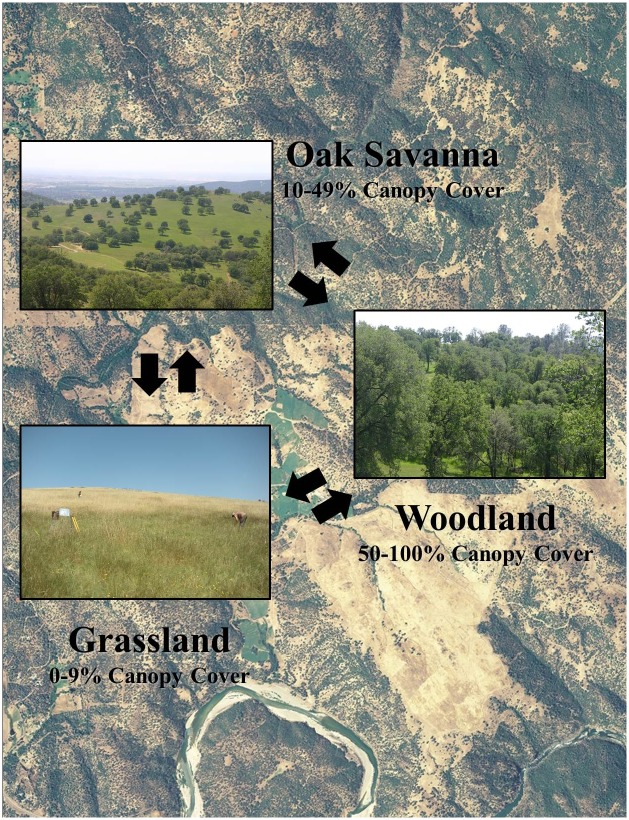
State and transition model for Sierra Nevada Gravelly Loam Foothill ecological site. A simplified expert state and transition model (STM) for the Sierra Nevada Sierra Nevada Gravelly Loam Foothill ecological site. Boxes represent vegetation states and the arrows represent thresholds between states. The underlying orthoimagery (USDA-NAIP imagery 2014) illustrates the gradient of oak woodland management across the study area. The images within the boxes provide an example visualization of the respective vegetative states. Photo credit: Danny Eastburn.

Here, we illustrate this approach using a managed oak (*Quercus* spp.) woodland landscape in California, USA. Globally, Mediterranean oak woodlands and savannas hold great social, ecological, and economic value, and are often the focus of conservation, restoration, and management efforts [19–21]. In California, oak woodlands—which are among the state’s richest and most highly valued ecosystems (Fig 1)—have undergone significant compositional and structural transformations [22]. The interactive effects of exotic species introductions, landscape engineering, climatic changes, and shifts in natural fire regimes have converted California’s native landscapes into a mosaic of alternative stable vegetation states that potentially deliver different levels and bundles of ecosystem services. Dominant states in this landscape are annual grassland with little to no woody canopy, partially-thinned oak savanna, and intact oak woodland which is the referent vegetation state before human intervention (Fig 2). We used multiple ecosystem service metrics to quantify the level of agricultural production, biodiversity and habitat, and soil health services delivered across these three dominant vegetation states (Fig 2). This allowed us to examine ecosystem service tradeoffs and synergies across the three vegetation states and scale up to a typical management unit to highlight potential economic and conservation outcomes. Quantifying tradeoffs and synergies between agricultural production and maintenance of other ecosystem services is a central need in moving toward management for multiple outcomes [1, 23].

## Methods

### Study area

This study was conducted at the University of California Sierra Foothill Research and Extension Center (SFREC) in Yuba County, California, US (Fig 1). SFREC has a Mediterranean climate with hot, dry summers and cool, wet winters. The research center is included within the Gravelly Loam Foothills ecological site of California’s Sierra Nevada Foothills—Major Land Resource Area 18 [24]. Common woody species include *Quercus douglasii* (blue oak), *Q*. *wislizenii* (live oak), *Pinus sabiniana* (foothill pine), *Ceanothus* spp. (buck brush and deer brush), and *Toxicodendron diversilobum* (poison oak). Understory vegetation is dominated by winter growing, annual grasses such as *Avena barbata* (wild oats), *Bromus hordeaceus* (soft chess), and *Bromus diandrus* (ripgut brome) and annual forbs such as *Erodium spp*. (filaree), and *Torilis arvensis* (hedge parsley) and legumes such as *Trifolium hirtum* (rose clover).

SFREC’s landscape is a mosaic of open grasslands, oak savannas, and woodlands—resulting from anthropogenic removal of woody plant cover (Fig 2). Across SFREC woody species were completely cleared in areas to create open grasslands during the 1960s, and selective partial stand thinning continued in other areas to create oak savannas until the 1980s, for forage improvement objectives. The resulting gradient of woody cover (non-thinned woodlands–partially thinned oak savannas–open grasslands) across this landscape has created a model natural laboratory in which to examine ecosystem service trade-offs and synergies between human-created, stable vegetation states.

### Study design and data collection

We used a cross-sectional survey approach to examine metrics of **agricultural production**, **biodiversity and habitat**, and **soil health** ecosystem services across three alternative, stable vegetation states. We established 600 m^2^ plots at 57 sites, which we randomly stratified across cattle grazed grassland, oak savanna, and oak woodland vegetation states [25, 26]. Each site had multiple 1 m^2^ plots established and caged to exclude grazers. The 600 m^2^ plots were divided into six 100m^2^ plots in which we used ocular estimates to determine percent coverage of each area type (open, interstitial, or closed canopy). The total estimated percentage of each area type (open, interstitial, or closed canopy) represented by each plot was used to calculate weighted averages for each ecosystem service metric at the respective site.

#### Vegetation states

We classified the 600 m^2^ sites into vegetation states based on the ocular estimation of canopy cover. We defined grassland as 0–9% canopy cover, oak savanna as 10% to 49% canopy cover, and oak woodland/forest as 50% to 100% canopy cover.

#### Metrics of agricultural production

During the 2010 and 2011 study years, we measured herbaceous annual net primary productivity (ANPP) at 255 total 1 m^2^ microplots across 57 sites. Forage production was measured at peak standing crop (representative of ANPP in annual grassland system) via the comparative yield-paired plot method [27]. For this study, we assumed all herbaceous species were edible and available for livestock consumption.

We calculated an agricultural use value for each vegetation state by calculating livestock grazing capacities from the 2010–2011 average ANPP, and applying the average agricultural lease rates (based on grazing capacity) as reported by California Department of Food and Agriculture for the region [28]. For each vegetation state we estimated total grazing capacity in terms of the amount of dry forage matter (approximately 354 kg) required to support one animal unit month (AUM; one mature 454 kg cow for 30 days) [29]. We then subtracted from this value the AUM equivalent of recommended residual dry forage matter (RDM; previous season’s herbaceous plant material left standing) for each vegetation state. The recommended levels were 336 kg RDM for woodland, 560 kg RDM for savanna, and 672 kg RDM for grassland, which are the minimum RDM levels to promote fall forage production of desirable species and maintain soil health [30]. We calculated agricultural use values as:

$$USD_{agricultural\;use\;value}=AUM_{grazing\;capacity}\cdot {\left(USD\;\cdot \;AUM^{-1}\right)}_{avg\;lease\;rate}$$

$$AUM_{grazing\;capacity}=\left(F_{net\;annual\;forage\;production}-RDM_{recommend\;standard\;for\;state}\right)\cdot {\left(354\;kg\right)}^{-1}$$

#### Metrics of biodiversity and habitat

During the 2010 and 2011 study years, we also measured plant community diversity and richness, native richness, and invasive plant richness and cover at 255 total plots across the 57 sites. We estimated cover by species using Daubenmire cover classes [31]. Each plant species was also assigned to a native, non-native, and invasive plant functional group [32]. We calculated plant species diversity (Shannon-Weiner Index) using species cover class midpoint [31]. Biodiversity plays a complex multi-layered role and is critical to underpinning ecosystem functions and processes that affect the supply of ecosystem services; however, biodiversity is often intrinsically valued and generally considered as a ‘supporting’ ecosystem service [1, 5, 33].

#### Metrics of soil health

During the 2010 study year, we intensively sampled a set of soil health metrics at 15 sites. We excavated and characterized multiple representative soil profiles (68 total soil profiles) associated with the 1-m^2^ plots. Soils were sampled from A and AB horizons, air dried and prepared for total C/N analysis using a dry combustion elemental analyzer[34]. Bulk density was measured using a 3D scanner method [35, 36].We measured surface infiltration at field capacity via a single-ring (39.7 cm inside diameter) falling head infiltrometer at each 1 m^2^ plot. We also calculated soil carbon pools for each vegetation state using horizon thickness, bulk density, percent rock fragment, and soil carbon concentration. Using settled auction prices from California’s cap-and-trade program administered by California Air Resources Board (CARB), we calculated the potential value of soil carbon stores as

$$USD_{CARB\;offset\;value}=\left(Mg\;CO_{2}ce_{soil\;C\;pool}-Mg\;CO_{2}ce_{\text{min}soil\;C\;pool}\right)\cdot {\left(USD\cdot Mg\;CO_{2}ce\right)}_{market\;value}$$

where ce is carbon equivalent, and ce_min_ is the minimum carbon equivalent from baseline conditions (i.e., grassland state) (www.arb.ca.gov/cc/capandtrade/auction/results_summary.pdf).

#### Scaling economic outcomes

We evaluated potential economic outcomes based on grazing revenue and carbon sequestration values for three scenarios of various compositions of vegetation states. The calculated grazing and carbon store values were scaled to 251 ha, which is the median area for a privately owned ranch in California [37]. We based the scenarios on three divergent land management strategies, the first scenario maximized agricultural production, the second scenario maximized soil carbon sequestration, and the third scenario explored a mosaic mixed land-use approach to balance agricultural and conservation outcomes at the ranch enterprise level. We chose such divergent scenarios to simply illustrate the potential range of responses.

### Data analysis

To examine differences in levels of ecosystems services delivered across alternative stable states, we tested relationships between vegetation state and each ecosystem service metric via linear regression and linear mixed effects analyses. To account for repeated sampling, plot ID was designated as grouping variables (random effects) [38, 39]. To account for possible mean differences between years, random effects for year were also included. Statistical analyses were conducted in Stata/SE 13. Standard diagnostic analyses were utilized to check distributional assumptions and homogeneity of variance.

## Results

**Fig 3:**
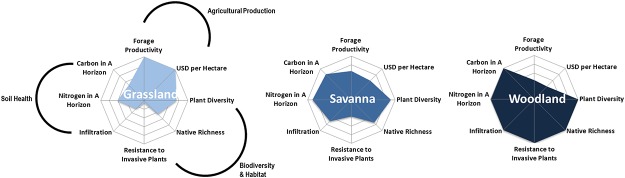
Multiple ecosystem service outcomes across vegetation states. Displays the provisioning of multiple ecosystem services across vegetation states. Values were relativized by maximum observed levels across all three vegetation states.

The level of agricultural production services, as measured by forage productivity and grazing value, was greatly enhanced in grassland compared to oak woodland states (Fig 3). Peak herbaceous production was significantly greater in grassland than in either savanna (*p* = 0.017) or oak woodland states (*p*<0.001) (Fig 3 and Table 1). Conversely, biodiversity and habitat and soil health service levels were lowest in grassland vegetation states (Fig 3). Grassland plant communities were less rich and less diverse than oak savanna and woodland understory herbaceous communities (Fig 3 and Table 1). Grassland communities also had greater numbers of highly invasive species and increased cover of *Taeniatherum canput-medusae*, which is a noxious highly invasive weed on western North American rangelands (Fig 3 and Table 1). The oak woodland vegetation state had the greatest understory plant diversity and native richness, lowest levels of invasion, and highest soil surface infiltration rates (Fig 3 and Tables 1 and 2). Calculated grazing capacities were 6.8 AUM ha^-1^ for grassland, 4.2 AUM ha^-1^ for savanna, and 2.8 AUM ha^-1^ for woodland vegetation states—which renders potential agricultural use values of 120 USD ha^-1^ for grassland, 74 USD ha^-1^for savanna, and 50 USD ha^-1^ for woodland. We calculated the soil organic carbon pools for the A horizon of each vegetation state as 20.98± 0.08 Mg C ha^-1^ for grassland, 27.5±0.18 Mg C ha^-1^ for savanna, and 32.4±0.13 Mg C ha^-1^ for woodland. The organic carbon pools for the AB horizon were calculated as 14.0±0.04 Mg C ha^-1^ for grassland, 25.5±0.04 Mg C ha^-1^ for savanna, and 23.8±0.03 Mg C ha^-1^ for woodland.

**Table 1:** Agricultural production, habitat and biodiversity.

	Grassland Mean (se)	Savanna Mean (se)	Woodland Mean (se)
Peak Herbaceous Biomass (g m^-2^)	309.3 (33.3)a	203.3 (23.2)b	132.9 (9.7)c
Species Richness (count)	11.7 (0.7)a	15.5 (0.8)b	16.5 (0.7)b
Plant Diversity (Hꞌ)	1.7 (0.1)a	2.1 (0.1)b	2.3 (0.1)c
Native Richness (count)	2.3 (0.3)a	4 (0.4)b	5.4 (0.5)c
Taeniatherum canput-medusae (% cover)	21.3 (3.0)a	9 (2.5)b	0.2 (0.1)c
Highly Invasive Richness (count)	1 (0.1)a	0.7 (0.1)b	0.1 (0.0)c

Pairwise comparisons for component metrics (n = 57) of agricultural production, and biodiversity and habitat ecosystem services. Within each ecosystem service metric, values with different letters are significantly different (p < 0.1).

**Table 2:** Soil health.

	Grassland Mean (se)	Savanna Mean (se)	Woodland Mean (se)
Infiltration (cm hr^-1^)	28.9 (4.6)a	76.1 (7.5)b	109.6 (18.0)c
A Horizon Total Nitrogen (g kg^-1^)	2.2 (0.2)a	3.1 (0.2)b	3.5 (0.3)b
A Horizon Total Carbon (g kg^-1^)	25.1 (2.9)a	41.4 (2.8)b	50.2 (4.5)c
AB Horizon Total Nitrogen (g kg^-1^)	0.7 (0.1)a	1.1 (0.1)b	1 (0.1)b
AB Horizon Total Carbon (g kg^-1^)	8.1 (1.1)a	13.8 (1.5)b	13.7 (1.0)b

Pairwise comparisons for ecosystem service metrics (n = 15) of soil health. Within each ecosystem service metric, values with different letters are significantly different (p < 0.1).

The three land-use scenarios yielded markedly different economic outcomes for this working landscape. In the scenario maximizing grazing value, the entire land resource base (251 ha) was assigned to open grassland—resulting in a potential annual agricultural lease revenue of 30,210 USD (Table 3). In the scenario maximizing carbon sequestration value, the land resource base was maintained as undisturbed woodland, which resulted in a potential annual opportunity cost of 17,770 USD or generating about 41% of the potential agricultural lease revenue. In third scenario, we assigned 50% of the land base to woodland and equally divided the remaining land resources to grassland and savanna, which resulted in a potential annual opportunity cost of 11,773 USD or about 61% of potential lease revenue.

**Table 3:** Landscape vegetation state composition management scenarios.

(a)	Grassland ha	Woodland ha	Savanna ha	Agricultural Revenue yr^-1^	
Maximize agricultural production scenario	251	0	0	30,210 USD	
Maximize conservation scenario	0	251	0	12,440 USD	
Balance agriculture and conservation services scenario	62.75	125.5	62.75	18,437 USD	
(b)	Grassland ha	Woodland ha	Savanna ha	Soil Carbon Pool in A & AB horizons (Mg)	Lifetime Offset Revenue
aximize agricultural production scenario	251	0	0	8782	0 USD
Maximize soil carbon storage scenario	0	251	0	14126	237,338 USD
Balance agriculture and conservation services scenario	62.75	125.5	62.75	12585	168,890 USD

Landscape vegetation state composition scenarios showcasing the economical outcomes of decisions emphasizing conservation, agricultural production and multiple goals at the ranch scale. (a) Illustrates the economic outcomes associated with an annual opportunity cost in terms of lost potential revenue. (b) The CARB Cap and Trade program offset revenue is calculated by using the value of the amount of carbon potentially stored over baseline.

## Discussion

We utilized a suite of dynamic vegetation and soil metrics within an STM framework to link management decisions with ecosystem service outcomes. Nation-wide, there is a growing need to integrate dynamic ecosystem service metrics—those indicators that change over human time scales and are largely induced by management—into conservation decision-making and assessments [1, 14]. Quantifying the benefits society derives from ecosystems and the potential tradeoffs among these benefits are key to increasing effectiveness of conservation programs and policies [2]. The oak woodland ecosystem has been commonly recognized for both its ecological and economic importance and provides a model system to explore a framework linking management decisions with multiple ecosystem service outcomes.

Our analysis reveals strong tradeoffs among the targeted agricultural and conservation outcomes for oak management practices (Fig 3) in this system. Agricultural losses, in terms of forgone increases in forage production, also mean large, annual opportunity costs at the ranch scale. The long-term cost (biodiversity and habitat and soil health services) of these tradeoffs (Figs 2 and 3) is made clear in the STM framework, which emphasizes breakpoints between states (i.e., thresholds) and the processes, energy, and inputs required to transition between states [14, 15]. Given the intensive inputs required for oak restoration success [22], conversion of woody habitats to open grassland likely will translate to permanent losses in conservation outcomes.

Restoration and conservation of oak woodland habitat has the potential to generate economic income from large stable soil carbon stores. Using our land-use scenarios as an example, a gain of 5,345 Mg C could be realized, over the baseline level of the soil C pool in a grassland vegetation state, by restoring or conserving (i.e., avoided conversion to grassland) 251 ha of oak woodland (Table 1). The California Cap and Trade Program November, 2014 auction settled at 12.10 USD for one metric ton of carbon dioxide equivalent (MTCO_2_e). This could potentially generate 237,338 USD (minus transaction and evaluation costs) through oak reforestation or avoided conversion projects for carbon emission offsets, if qualified under California Air Resources Board approved offset registry.

Our results reveal an opportunity to increase the implementation of land management practices supporting multiple ecosystem service goals. One potential avenue is to foster research and management collaborations to investigate opportunities for compatibilities and win-win benefits for both agricultural production and environmental outcomes. Identifying these win-win bundles of ecosystem services may enhance the efficiency of PES markets. Ecosystem service markets or payments for ecosystem services are also potential options to assist ranchers with management tradeoffs in meeting the economic realities of agricultural production. Investments in conservation easement programs and cost sharing for conservation practices are a few existing opportunities that could be further developed.

Our analysis also highlights the contributions of alternative stable vegetation states to different bundles of ecosystem services (Fig 3), and points to potential benefits in maintaining landscape heterogeneity to optimize agricultural and conservation goals. However, even in a ‘balanced’ scenario, there are long-term costs of foregone annual revenues (Table 3). Mainstreaming conservation programs on working landscapes will require inclusion of offsets (e.g., via payments for other ecosystem services provided) for forgone agricultural revenue. This work highlights ecosystem service potential metrics that could be integrated into payments for ecosystem service programs or markets.

## Conclusions

This is a quantitative demonstration of how STM frameworks can be used to link management decision with on-the-ground ecosystem service metrics and quantify tradeoffs and synergies among agricultural production and conservation outcomes. This work highlights the benefits of using a mix of vegetative states across the landscape to support both agricultural and conservation goals, and provides a working example of how STMs can be used in developing conservation program incentives. Integrating measurable, dynamic ecosystem service indicators into management models will be valuable to both landowners interested in managing for multiple outcomes, and agencies responsible for assessing conservation practices and allocating funds. These tools will also be key in assessing potential threats from a changing environment, including increasing severity and frequency of droughts, increasing wildfire frequency and duration, and land-use changes.

## References

[1] MEA. Millennium ecosystem assessment, ecosystems and human well-being: synthesis. Island Press, Washington, DC; 2005.

[2] Briske DD. Conservation benefits of rangeland practices: assessment, recommendations, and knowledge gaps. Lawrence, Kansas, USA: United States Department of Agriculture, Natural Resources Conservation Service; 2011 429 p.

[3] Claassen R. USDA conservation spending on working agricultural lands bucks long-term trend. Amber Waves 2011:9–60.

[4] Goldstein JH, Presnall CK, López-Hoffman L, Nabhan GP, Knight RL, Ruyle GB, et al Beef and Beyond: Paying for Ecosystem Services on Western US Rangelands. Rangelands. 2011;33(5):4–12.

[5] Havstad KM, Peters DPC, Skaggs R, Brown J, Bestelmeyer B, Fredrickson E, et al Ecological services to and from rangelands of the United States. Ecol Econ. 2007;64(2):261–8.

[6] Huntsinger L, Johnson M, Stafford M, Fried J. Hardwood Rangeland Landowners in California from 1985 to 2004: Production, Ecosystem Services, and Permanence. Rangel Ecol Manag. 2010;63(3):324–34.

[7] Merenlender AM, Huntsinger L, Guthey G, Fairfax SK. Land trusts and conservation easements: Who is conserving what for whom? Conserv Biol. 2004;18(1):65–75.

[8] TEEB. The Economics of Ecosystems and Biodiversity: Ecological and Economic Foundations. Kumar P, editor. London and Washington: Earthscan; 2010.

[9] Kremen C. Managing ecosystem services: what do we need to know about their ecology? Ecol Lett. 2005;8(5):468–79. 10.1111/j.1461-0248.2005.00751.x 21352450

[10] Shaw MR, Pendleton L, Cameron DR, Morris B, Bachelet D, Klausmeyer K, et al The impact of climate change on California's ecosystem services. Clim Change. 2011;109:465–84.

[11] Bennett EM, Peterson GD, Gordon LJ. Understanding relationships among multiple ecosystem services. Ecology letters. 2009;12(12):1394–404. 10.1111/j.1461-0248.2009.01387.x 19845725

[12] Carpenter SR, DeFries R, Dietz T, Mooney HA, Polasky S, Reid WV, et al Millennium Ecosystem Assessment: Research Needs. Science. 2006;314(5797):257–8. 10.1126/science.1131946 17038608

[13] Rudd MA, Fleishman E. Policymakers' and Scientists' Ranks of Research Priorities for Resource-Management Policy. Bioscience. 2014;64(3):219–28.

[14] Brown J, MacLeod N. A site-based approach to delivering rangeland ecosystem services. The Rangeland Journal. 2011;33(2):99–108. 10.1071/RJ11006.

[15] Briske DD, Fuhlendorf SD, Smeins EE. State-and-transition models, thresholds, and rangeland health: A synthesis of ecological concepts and perspectives. Rangeland Ecology & Management. 2005;58(1):1–10.

[16] Westoby M, Walker B, Noy-Meir I. Opportunistic Management for Rangelands Not at Equilibrium. Journal of Range Management. 1989;42(4):266–74.

[17] Twidwell D, Allred BW, Fuhlendorf SD. National-scale assessment of ecological content in the world's largest land management framework. Ecosphere. 2013;4(8):art94.

[18] Rumpff L, Duncan DH, Vesk PA, Keith DA, Wintle BA. State-and-transition modelling for Adaptive Management of native woodlands. Biol Conserv. 2011;144(4):1224–36. 10.1016/j.biocon.2010.10.026.

[19] Bugalho MN, Caldeira MC, Pereira JS, Aronson J, Pausas JG. Mediterranean cork oak savannas require human use to sustain biodiversity and ecosystem services. Front Ecol Environ. 2011;9(5):278–86.

[20] Roche LM, Rice KJ, Tate KW. Oak conservation maintains native grass stands in an oak woodland-annual grassland system. Biodivers Conserv. 2012;21(10):2555–68.

[21] Marañón T, Pugnaire FI, Callaway RM. Mediterranean-climate oak savannas: the interplay between abiotic environment and species interactions. Web Ecology. 2009;9:30–43.

[22] McCreary D. A quarter century of oak woodland research in the Sierra foothills supports oak restoration. California Agriculture. 2010;64(2):63–8.

[23] Raudsepp-Hearne C, Peterson GD, Bennett EM. Ecosystem service bundles for analyzing tradeoffs in diverse landscapes. Proceedings of the National Academy of Sciences. 2010;107(11):5242–7. 10.1073/pnas.0907284107 PMC2841950 20194739

[24] Ecological site description (ESD) system for rangeland and forestland data. [Internet]. 2011.

[25] Keeley JE, Fotheringham CJ. Plot shape effects on plant species diversity measurements. Journal of Vegetation Science. 2005;16(2):249–56.

[26] Keeley JE, Fotheringham CJ. Species–area relationships in Mediterranean-climate plant communities. Journal of Biogeography. 2003;30(11):1629–57.

[27] Interagency. Utilization studies and residual measurements Interagency technical reference BLM/RS/ST-96/004+1730. Denver, Colorado, USA: USDI Bureau of Land Management's National Applied Resources Science Center; 1996.

[28] CDFA. California agricultural statistics review 2013–2014. Sacramento, CA, USA: California Department of Food and Agriculture; 2014.

[29] Holechek J, Pieper R, Herbel C. Range management, principles and practices. Englewood Cliffs, New Jersey, USA: Prentice Hall; 1989 456 p.

[30] Bartolome JW, Frost WE, McDougald NK, Connor M. California guidelines for residual dry matter (RDM) management on coastal and foothill annual rangelands: University of California, Division of Agriculture and Natural Resources; 2002.

[31] Bonham CD. Measurements for terrestrial vegetation. New York, New York, USA: John Wiley and Sons; 1989 338 p.

[32] DiTomaso JM, Healy EA. Weeds of California and other western states. Oakland, California, USA: Regents of the University of California Division of Agriculture and Natural Resources; 2007 834 p.

[33] Mace GM, Norris K, Fitter AH. Biodiversity and ecosystem services: a multilayered relationship. Trends Ecol Evol. 2012;27(1):19–26. 10.1016/j.tree.2011.08.006 21943703

[34] Costech Analytical Technologies Inc. Total C/N analysis using a dry combustion elemental analyzer Valencia, CA: Costech Analytical Technologies Inc.

[35] Rossi AM, Hirmas DR, Graham RC, Sternberg PD. Bulk Density Determination by Automated Three-Dimensional Laser Scanning. Soil Science Society of America Journal. 2008;72(6):1591–3.

[36] Inc. N. NextEngine DeskTop 3D Scanner Model 2020i. Santa Monica, CA.

[37] Roche LM, Schohr TK, Derner JD, Lubell MN, Cutts BB, Kachergis E, et al Sustaining Working Rangelands: Insights from Rancher Decision Making. Rangel Ecol Manag. 2015;68(5):383–9.

[38] Rabe-Hesketh S, Skrondal A. Generalized linear mixed-effects models. Ch Crc Handb Mod Sta. 2009:79–106.

[39] Pinheiro J, Bates D. Mixed-efi'ects models in S and S-PLUS Statistics and Computing, Springer-Verlag, New York 2000.

